# Supplementary material for: Proteomic and Transcriptomic Landscapes of Alström and Bardet–Biedl Syndromes
Source: Genes (Basel). 2022 Dec 15;13(12):2370. doi: 10.3390/genes13122370 (PMC9777683; doi:10.3390/genes13122370)
Supplement: Supplementary file 1 [file genes-13-02370-s001.zip › genes-2032606-supplementary/Supplementary tables.pdf]

Table S1. List of significantly up- and downregulated transcripts in ALMS and BBS.

upregulated in ALMS (FC > 1.5)	upregulated in BBS (FC > 1.5)	downregulated in ALMS (FC < 0.67)	downregulated in BBS (FC < 0.67)
TRIM4	*TRIM4*	*ZIC1*	*PTH1R*
HIST1H3C	*HIST1H3C*	*PTH1R*	*ZIC1*
CCDC152	*CCDC152*	*STMN2*	*PAX7*
CTCFL	*HIST1H4L*	*PAX7*	*STMN2*
HIST1H4L	*ZNF558*	*CNTFR*	*SCGB3A2*
ZNF558	*TEK*	*FOXA1*	*CR1L*
HTR7	*HMOX1*	*SCGB3A2*	*MECOM*
SGIP1	*HTR7*	*OLIG3*	*CNTFR*
VSIG1	*SGIP1*	*PAX6*	*HAPLN1*
IFNA16	*VSIG1*	*RSPO3*	*DPP6*
ENPEP		*PITX2*	*RSPO3*
		SLITRK2	*PAX6*
		MECOM	*GTF2A1L*
		NPFFR2	*FOXA1*
		DKK1	*TBX1*
		CR1L	*OLIG3*
		HAPLN1	*PITX2*
		SCNN1G	*LOC101927668*
		SCD5	*SCD5*
		TBX1	*TENM1*
		SP8	*TFAP2C*
		TFAP2C	*SP8*
		ARSE	*ARSE*
		SLC16A12	*CACNA2D3*
		TMEM88	*TMEM88*
		CHRNA3	*NRG3*
		LOXL3	*SCNN1G*
		SORBS2	*NTRK3*

		DMBX1<br>*DLL1*<br>*ROR2*<br>*CHRNB4*<br>*TMEM51*<br>*TMEM150C*<br>*ZADH2*	*MCOLN3*<br>*GABRB2*<br>*NR5A2*<br>*NPFFR2*<br>*SORBS2*<br>*ADGRA2*<br>*DLL1*<br>*VIPR2*<br>*CHRNA3*<br>*ZFHX4*<br>*DMBX1*<br>*SEZ6*<br>*POTEF*

Table S2. List of significantly up- and downregulated proteins in ALMS and BBS.

upregulated in ALMS (FC > 1.5)	upregulated in BBS (FC > 1.5)	downregulated in ALMS (FC < 0.67)	downregulated in BBS (FC < 0.67)
CRYZ	CRYZ	MYH14	IFITM3
CGREF1	DYNC2LI1	HSD11B2	GPC4
CDC25C	NAB2	TMEM63A	MBP
FAH	APOBEC3B	ALMS1	CLN8
ASAH1	LGALS1	GALNT3	SOX3
HSBP1	FABP7	IFITM3	
CDKN2AIPNL	SELENBP1	ITPR2	
AGGF1	FHL1	ZSCAN10	
C9orf64	HEBP1	GPC4	
TCEAL5	KIF17	METTL7A	
NAB2	TRIM5	ARRB1	
ARHGAP8	DERA	POU5F1	
	SCRN2	CD9	
	DDAH2		
	CYBA		
	DMXL2		

Table S3. List of reactome pathways significantly up- and down-regulated by both proteomic and transcriptomic analysis in ALMS and BBS syndromes. P-proteomics; T- transcriptomics.

Reactome Pathway	ALMS		BBS	
	P	T	P	T
RRNA_MODIFICATION_IN_THE_NUCLEUS_AND_CYTOSOL	↓	↑	↓	↑
MITOCHONDRIAL_TRANSLATION<br>TRANSPORT_OF_MATURE_TRANSCRIPT_TO_CYTOPLASM<br>TRANSLATION			↓	↑
STABILIZATION_OF_P53<br>REGULATION_OF_PTEN_STABILITY_AND_ACTIVITY<br>CROSS_PRESENTATION_OF_SOLUBLE_EXOGENOUS_ANTIGENS_ENDOSOMES_<br>DECTIN_1_MEDIATED_NONCANONICAL_NF_KB_SIGNALING<br>DNA_REPLICATION<br>G2_M_CHECKPOINTS<br>ASSEMBLY_OF_THE_PRE_REPLICATIVE_COMPLEX<br>DNA_REPLICATION_PRE_INITIATION	↑	↑	↑	↑
AUF1_HNRNP_D0_BINDS_AND_DESTABILIZES_MRNA<br>REATOME_APC_C_CDH1_MEDIATED_DEGRADATION_OF_CDC20_AND_OTHER_APC_C_CDH1_TARGETED_PROTEINS_IN_LATE_MITOSIS_EARLY_G1<br>SWITCHING_OF_ORIGINS_TO_A_POST_REPLICATIVE_STATE<br>METABOLISM_OF_POLYAMINES<br>DNA_STRAND_ELONGATION<br>DEGRADATION_OF_GLI1_BY_THE_PROTEASOME<br>APC_C_MEDIATED_DEGRADATION_OF_CELL_CYCLE_PROTEINS<br>ORC1_REMOVAL_FROM_CHROMATIN<br>S_PHASE<br>DEGRADATION_OF_DVL<br>FCERI_MEDIATED_NF_KB_ACTIVATION<br>NEGATIVE_REGULATION_OF_NOTCH4_SIGNALING<br>DOWNSTREAM_SIGNALING_EVENTS_OF_B_CELL_RECEPTOR_BCR_<br>CELLULAR_RESPONSE_TO_HYPOXIA<br>DEGRADATION_OF_AXIN<br>CELL_CYCLE_CHECKPOINTS<br>REGULATION_OF_RAS_BY_GAPS<br>UCH_PROTEINASES<br>REGULATION_OF_RUNX3_EXPRESSION_ANDACTIVITY<br>MITOTIC_G1_PHASE_AND_G1_S_TRANSITION<br>HIV_INFECTION<br>REGULATION_OF_MRNA_STABILITY_BY_PROTEINS_THAT_BIND_AU_RICH_ELEMENTS<br>HOST_INTERACTIONS_OF_HIV_FACTORS<br>THE_ROLE_OF_GTSE1_IN_G2_M_PROGRESSION_AFTER_G2_CHECKPOINT<br>G2_M_DNA_DAMAGE_CHECKPOIN<br>G1_S_DNA_DAMAGE_CHECKPOINTS<br>ACTIVATION_OF_ATR_IN_RESPONSE_TO_REPLICATION_STRESS<br>RESOLUTION_OF_AP_SITES_VIA_THE_MULTIPLE_NUCLEOTIDE_PATCH_REPLACEMENT_PATHWAY<br>LAGGING_STRAND_SYNTHESIS<br>PCNA_DEPENDENT_LONG_PATCH_BASE_EXCISION_REPAIR<br>SEPARATION_OF_SISTER_CHROMATIDS<br>CYCLIN_A_CDK2_ASSOCIATED_EVENTS_AT_S_PHASE_ENTRY<br>SIGNALING_BY_THE_B_CELL_RECEPTOR_BCR_<br>ANTIGEN_PROCESSING_UBIQUITINATION_PROTEASOME_DEGRADATION<br>INTERCONVERSION_OF_NUCLEOTIDE_DI_AND_TRIPHOSPHATES<br>DNA_REPAIR<br>PROCESSIVE_SYNTHESIS_ON_THE_LAGGING_STRAND<br>CELL_CYCLE_MITOTIC<br>MISMATCH_REPAIR<br>INTERLEUKIN_1_SIGNALING			↑	↑

MAPK6_MAPK4_SIGNALING<br>TELOMERE_C_STRAND_LAGGING_STRAND_SYNTHESIS<br>UB_SPECIFIC_PROCESSING_PROTEASES<br>SCF_SKP2_MEDIATED_DEGRADATION_OF_P27_P21<br>PROCESSING_OF_DNA_DOUBLE_STRAND_BREAK_ENDS<br>DEGRADATION_OF_BETA_CATENIN_BY_THE_DESTRUCTION_COMPLEX<br>GAP_FILLING_DNA_REPAIR_SYNTHESIS_AND_LIGATION_IN_GG_NER<br>RECOGNITION_OF_DNA_DAMAGE_BY_PCNA_CONTAINING_REPLICATION_COMPLEX<br>NUCLEOTIDE_SALVAGE<br>METABOLISM_OF_NUCLEOTIDES<br>DEUBIQUITINATION<br>NEDDYLATION<br>CLEC7A_DECTIN_1_SIGNALING<br>RESOLUTION_OF_ABASIC_SITES_AP_SITES_<br>BUDDING_AND_MATURATION_OF_HIV_VIRION<br>INTRAFLAGELLAR_TRANSPORT<br>ENDOSOMAL_SORTING_COMPLEX_REQUIRED_FOR_TRANSPORT_ESCRT_				
TRANSCRIPTIONAL_REGULATION_OF_PLURIPOTENT_STEM_CELLS<br>SLC_MEDIATED_TRANSMEMBRANE_TRANSPORT	↓	↓	↓	↓
TRANSPORT_OF_VITAMINS_NUCLEOSIDES_AND_RELATED_MOLECULES	↓	↓		
ION_TRANSPORT_BY_P_TYPE_ATPASES<br>NCAM1_INTERACTIONS<br>GLYCOSAMINOGLYCAN_METABOLISM<br>CALNEXIN_CALRETICULIN_CYCLE<br>SYNDECAN_INTERACTIONS<br>TRANSPORT_OF_BILE_SALTS_AND_ORGANIC_ACIDS_METAL_IONS_AND_AMINE_COMPOUNDS<br>A_TETRASACCHARIDE_LINKER_SEQUENCE_IS_REQUIRED_FOR_GAG_SYNTHESIS<br>CHONDROITIN_SULFATE_DERMATAN_SULFATE_METABOLISM<br>HEPARAN_SULFATE_HEPARIN_HS_GAG_METABOLISM<br>ER_QUALITY_CONTROL_COMPARTMENT_ERQC_<br>DISEASES_ASSOCIATED_WITH_GLYCOSAMINOGYCAN_METABOLISM<br>N_GLYCAN_TRIMMING_IN_THE_ER_AND_CALNEXIN_CALRETICULIN_CYCLE<br>HS_GAG_DEGRADATION<br>SYNTHESIS_OF_VERY_LONG_CHAIN_FATTY_ACYL_COAS<br>DEFECTIVE_B4GALT7_CAUSES_EDS_PROGEROID_TYPE<br>FATTY_ACYL_COA_BIOSYNTHESIS<br>HS_GAG_BIOSYNTHESIS			↓	↓
